# Initiation of postpartum modern contraceptive methods: Evidence from Tanzania demographic and health survey

**DOI:** 10.1371/journal.pone.0249017

**Published:** 2021-03-25

**Authors:** Martin M. Rwabilimbo, Bilikisu R. Elewonibi, Mashavu H. Yussuf, Masanja Robert, Sia E. Msuya, Michael J. Mahande

**Affiliations:** 1 Department of Epidemiology & Biostatistics, Institute of Public Health, Kilimanjaro Christian Medical University College, Moshi, Tanzania; 2 Bukoba Regional Referral Hospital, Bukoba, Tanzania; 3 Harvard T.H Chan School of Public Health, Boston, MA, United States of America; 4 Mwenge Catholic University (MWECAU), Moshi, Tanzania; University of Western Australia, AUSTRALIA

## Abstract

**Background:**

Postpartum contraceptive use is vital to improve maternal and child survival. It helps to have optimal child spacing, prevent unplanned pregnancies and associated adverse birth outcomes. However, postpartum contraceptive use in Tanzania remains low. Short median interval for resumption to sex after birth among African women has been associated with adverse maternal and child health wellbeing. This study aimed to assess optimal time to contraceptive use and predictors of time to contraceptive use after birth among women of reproductive age in Tanzania.

**Methods:**

A cross section study using the TDHS 2015–16 data was used. A total of 3775 postpartum women were analyzed. Information on pregnancy, births and contraceptive use were recorded over the previous 5 years with the focus on most recent birth from the contraceptive calendar. Data analysis was performed using Stata 14.0. Analysis accounted for complex survey design. Time to modern contraceptive use after birth was computed using Kaplan Meier estimate. Adjusted time ratios with 95% CI were estimated using Weibull accelerated failure time models.

**Results:**

A total weighted sample of 3775 women was analyzed. The median time to contraceptive use after birth was 7(IQR: 4–13) months, while for resumption to sex afterbirth was 2(IQR: 1–5) months. Factors such as never been married (TR: 1.63; 95%CI: 1.26–2.11) and breastfeeding (TR: 5.50; 95%CI: 4.12–7.35) were associated with longer time to postpartum contraceptive use. Belonging to richest wealth quintile (TR: 0.73; 95%CI: 0.54–0.99) and adopting long acting methods (TR: 0.70; 95%CI: 0.60–0.82) increased women’s likelihood of having shorter time to postpartum contraceptive use.

**Conclusion:**

There was a time lag of five months from resumption of sex and initiation of postpartum contraception use. The interceptive measures to facilitate timely availing methods of women’s choice and promotion of utilization of maternal health care services may reduce delays in postpartum contraceptive use.

## Introduction

Postpartum contraceptive use is important to improve maternal and child survival [1]. It prevents unplanned pregnancies, enhance optimal child spacing and has been attributed with 44% reduction in maternal mortality and 21% for under five mortality in low resourced countries [1–4]. Postpartum contraceptive use also helps the couples and individuals realize their basic right to decide autonomously when and how many children to have, participate in economic activities and education achievement [5].

Postpartum modern contraceptive prevalence in low and middle income countries has been reported to vary from 8% at first month,25% at sixth month to 30% at twelve months after birth [6]. In Tanzania, it varies from 3% at first month to 28% at one year after birth [6]. This figure is far below the national target of 45% (One Plan II) and 60% (health sector strategic plan IV) [7, 8]. Despite the country commitment to increase universal accessibility to family planning methods through improving commodity coverage, training providers, integration of postpartum contraceptive use to other maternal and child health services and PMTCT services [11, 12]. Still the unmet needs for postpartum modern contraceptive use remains higher as 81% before 6 months to 50% between 12–23 months [9].

The World Health Organization recommended to support postpartum women and advice to adopt modern contraceptives within six weeks postpartum [10]. However, numerous factors have been reported as barriers to modern contraceptive use in sub-Saharan countries including Tanzania. These include limited choice and access of methods, poor quality of available services, user and provider bias, fear or experience of side effects, myths and misconception about the contraception [11, 12].

Previous studies have reported short median interval for resumption to sex after birth among African women may affect maternal and child health wellbeing [13, 14]. These studies also indicated that factors such as education age, area of residence, level of empowerment, wealth quintile, antenatal care and postnatal care were associated with postpartum modern contraceptive use among married women in low income countries [15–18].

Delay or non-use of postpartum modern contraceptive have been associated with suboptimal interpregnancy interval and unintended pregnancies [19, 20]. In Tanzania, 47% of all pregnancies occur during sub-optimal/short interpregnancy interval of at least 24 months [9]. Short interpregnancy interval has been associated with an increased risk of an induced abortions, miscarriage, premature rupture of membranes, preterm births, utero-placental bleeding, small for gestation age, negative neurodevelopment outcomes, neonatal and child mortalities, stillbirths, and maternal depletion syndrome [21, 22]. Little have been documented on time to contraceptive use and its predictors among postpartum women in Tanzania. This study aimed to determine the time to modern contraceptive use and its predictor’s among postpartum women in Tanzania.

## Materials and methods

### Setting and data source

This was analytical cross-section study design which was conducted using secondary data from Tanzania demographic and Health Survey 2015–16 (TDHS 2015–16). The TDHS 2015–16 collected for the entire country (Mainland and Zanzibar Island) with intention to provide reliable and up-to-dated estimates on demographic and health indicators. These indicators included family planning, fertility levels and preferences, maternal mortality, infant and child mortality, nutritional status of mothers and children, antenatal care, delivery care, and childhood immunizations and diseases.

The TDHS 2015–16 used two-stage clustered sampling. During the first stage involved selecting 608 clusters consisting of enumeration areas (EAs) delineated for the 2012 Tanzania Population and Housing Census. In the second stage, 22 households were then systematically selected from each cluster, yielding a representative probability sample of 13,376 households for the 2015–16 TDHS. To estimate geographic differentials for certain demographic indicators, Tanzania was divided into nine geographic zones though not official administrative areas, this classification system is also used by the Reproductive and Child Health Section of the Ministry of Health [23]. Grouping the regions into zones allowed a relatively large number of people in the denominator and a reduced sampling error. Zones in this DHS include; Western Zone, Northern Zone, Central Zone, Southern Highlands Zone, Southern Zone, South West Highlands Zone, Lake Zone, Eastern Zone and Zanzibar. The survey included women aged 15–49 who were either usual residents or visitors in the household on the night before the survey. The standardized woman’s questionnaire and contraceptive calendar for eligible women was adapted to reflect the population and health issues relevant to Tanzania.

### Study population

**Fig 1:**
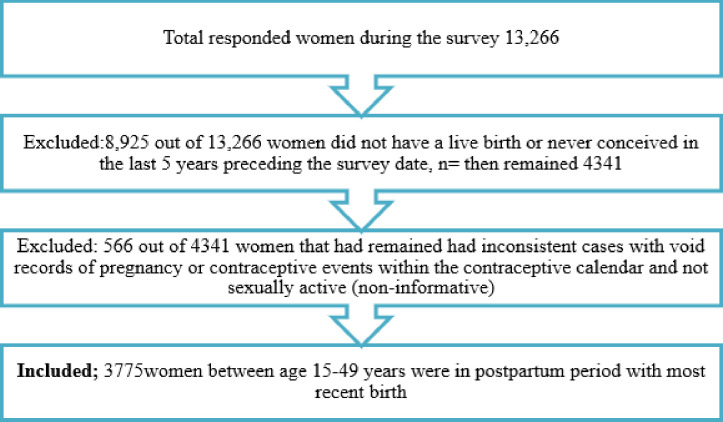
Sampling scheme.

This study included only sexually active women with the most recent birth that occurred 59 months preceding the survey dates even if a woman delivered more than once during the reference period. This was due to the fact that the DHS collects some maternal health care information (on antenatal, delivery, and postnatal care) for only the most recent birth. Study excluded women who did not resume sex after birth during that period and women with void or inconsistencies regarding the outcome. Further information can be obtained on the schematic diagram attached (Fig 1).

### Study variables and definitions

#### Dependent variable

Time to contraceptive use was the main outcome of interest. It was defined as the time interval taken by a woman after giving birth until she initiates the use of modern contraception given a woman resumed sex. The modern contraception initialization at less than 2months was considered as shorter time while adoption of the methods at 2 months or after was considered as longer time. The time to contraceptive use was computed from the contraceptive calendar which collects retrospective information regarding reproductive contraceptive use on monthly basis for the period of five years prior date of survey [24].

The status of postpartum contraceptive use (PPFP) was a binary variable coded 1 for a woman who used any contraceptive method (pill, IUD, injection, diaphragm, condom (male or female), sterilization (male or female), implant, or foam/jelly) and 0if otherwise after a live birth. The Lactational Amenorrhea Method (LAM) was to be excluded from the modern contraception category, as it is intended for only the first six months after childbirth; its use for the entire 24-months postpartum period is not advisable as the only method to depend on due to reduced effectiveness. Women were categorized as Long- acting users if they were using either of the following; reversible contraceptives (LARCs) such as intrauterine devices (IUDs) and hormonal implants or Permanent Methods such as male and female sterilization. On other hand short acting consisted of pills, male and female condoms, the standard days Method, diaphragms, spermicides and Injectables (although injectables are effective for up to 3 months, they were classified as a short-acting method, as is the norm). Traditional methods included withdrawal, periodic abstinence, and fork methods and were not included to be compared.

#### Independent variables

**Fig 2:**
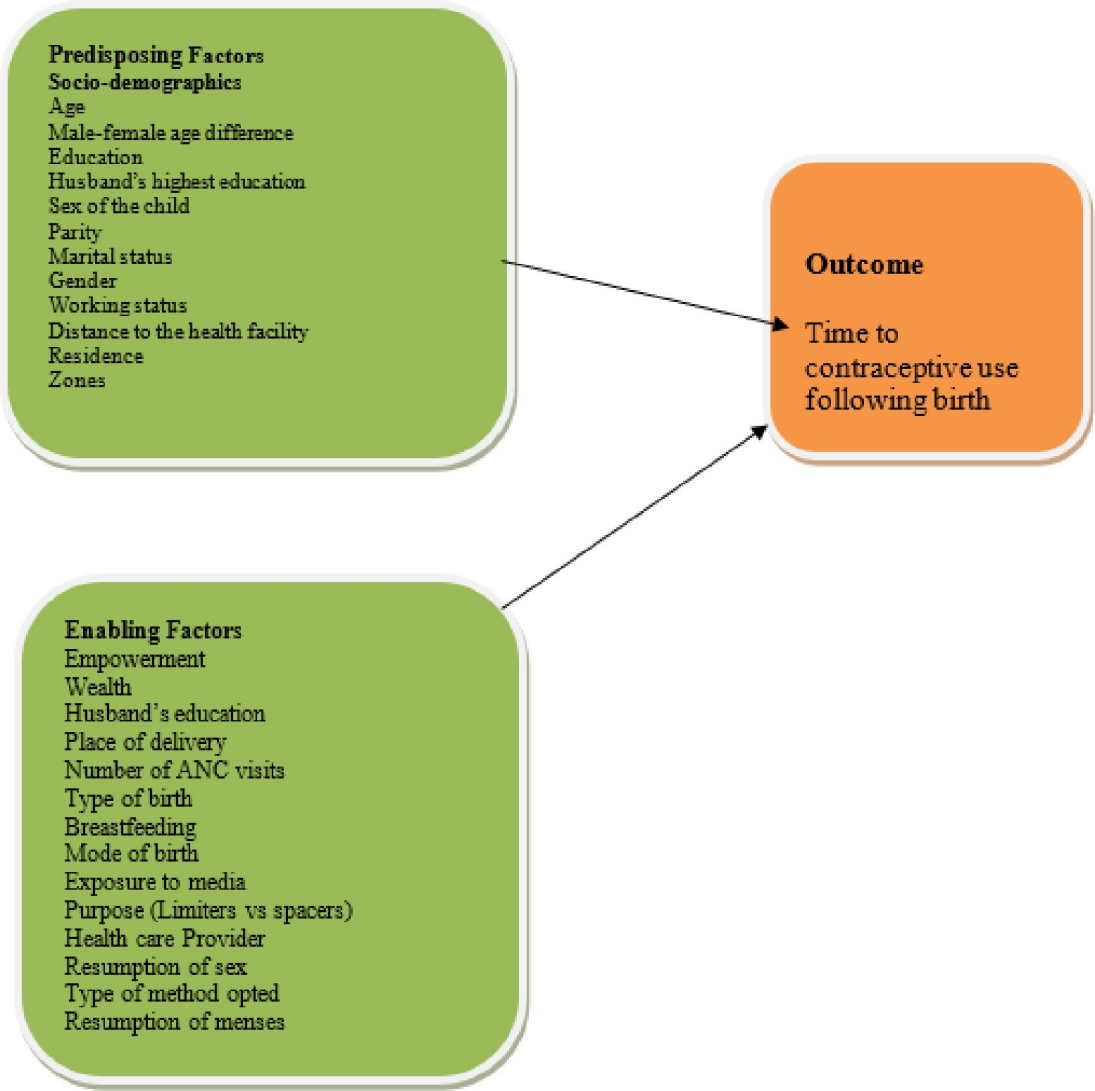
Conceptual framework on predictors of time to initiate postpartum contraceptive.

The independent variables include women empowerment, breastfeeding practices, antenatal visits, mode of delivery, and place of delivery postnatal care and exposure to media as enabling factors. Socio-demographics were age, area of residence, education, zones, husband’s education, and number of children ever born, wealth status, and marital status. Women empowerment was generated as an index using principal component analysis from woman’s decision making about contraception, household purchases, visits of family or relatives and woman’s earnings and further categorized into low, medium and highly empowered. Similarly, exposure to media was generated as a composite variable based on whether a woman accesses contraception information from newspapers, radio, and television or through internet followed by a binary categorization as whether exposed or not. Other factors included were receiving birth care from skilled health care provider, resumption of sex after recent birth, Fertility intentions, resumption of menses and type of method opted based on its duration of effectiveness. Furthermore, based on the purpose they were classified as limiters and spacers while based on the intentions expressed for contraception either intended or didn’t. Conceptual framework regarding the relationship can be seen on below (Fig 2). Further description of the variables can be obtained on the recode manual for DHS.

### Statistical analysis

Data analysis was performed using Stata version 14.0. Data were checked for inconsistence or missing values, duplicate entries prior the analysis. Data was set to account for both survey nature of the data and survival analysis using both “svy” and “stset” commands. Accounting for survey ensured representativeness and correction for non-responses while survival analysis was based on dependent variable nature being time. Descriptive statistics were summarized using frequency and proportions for categorical variables while mean and standard deviation as well as median and inter-quartile range (IQR) were used to summarize the continuous variables. An assessment of the time-to-contraceptive use after birth was computed using life tables and Kaplan Meier estimate. Survival curves and Wilcoxon log-rank test were used to estimate the difference in time to contraceptive use by participant characteristics. A non-informative censoring was assumed for the participants who did not start using any modern method. Weibull Accelerated Failure Time (Weibull AFT) modeling the survival time was used to estimate time ratios to postpartum modern contraceptive use across exposures. A P value of less than 0.05 was considered statistically significant.

The Time to event approach was proper as it allowed to deal with women irrespective of whether or not used modern contraceptive by the time of the survey (censoring) [25]. Model diagnostics for goodness of fit was also performed. If the plotted graphs did not yield parallel curves separated *β* over the log(time), then the proportion hazard (PH) assumption was considered violated. Multicollinearity was assessed using variance inflation factors (VIF), where variables with VIF >3 were considered collinear (34 and 31). Thus, empowerment and ANC visits were highly collinear, and therefore empowerment was removed from the model. Furthermore, variable expressing their intentions to use had few observations and hence despite its statistical significance in the crude analysis, this variable was not included in the model as would lower the power of the model(n = 1,176).

The approach for estimating time to modern contraceptive use with AFT model represented substantial improvement over approach used previously. It allowed making formal inference based on the survival time rather than hazard function and being parametric assumes distribution for the survival time’s, hence more accurate statistical inference, proper fitting of the model and easy interpretation of the results [25, 26]. Results for crude and adjusted analysis are presented in the form of time ratios for postpartum modern contraceptive use.

### Ethical considerations

Permissions to use the TDHS 2015–16 data was granted from the DHS MEASURE website. The study approval was obtained from KCMUCo Research and Ethical Review committee (CREC no 2410). It is worth knowing that during the survey DHS measure program ensures ethical conduct in accordance to human subject research.

## Results

### Socio-demographic characteristics of study participant’s

A total of 4341women were identified as postpartum women. Of these, 3775 were analyzed. This corresponds to a response rate of 87%. Characteristics of the study participants are shown in Table 1. Majority 2,981(78%) were married approximately two thirds of postpartum women had attained primary education level.

**Table 1:** Socio-demographic characteristics of the study participant (N = 3775).

Characteristics	n (%)	Weighted %
**Age**		
15–19	149(3.95)	4.34
20–24	735(19.47)	19.99
25–29	823(21.80)	22.45
30–34	728(19.28)	18.51
35–39	649(17.19)	16.46
40–44	447(11.84)	11.71
45–49	244 (6.46)	6.54
Mean age (SD)	30.95(8.22)	
**Partner age difference**		
Same age	123(3.26)	4.35
Man older ≤ 9 years	2,067(54.75)	68.82
Man older ≥ 9years	648(17.17)	2.24
Woman older	143 (3.79)	4.43
Missing	794(21.03)	
**Marital status**		
Never married	237(6.28)	6.92
Married /in union	2,981(78.97)	78.09
Separated/widowed/divorced	557(14.75)	14.99
**Residence**		
Urban	1,312(34.75)	38.37
Rural	2,463 (65.25)	61.63
**Zones**		
Western zone	249(6.60)	6.94
Northern zone	420(11.13)	12.62
Central zone	392(10.38)	11.28
Southern Highlands	450 (11.92)	8.36
Southern zone	340(9.01)	7.96
South West Highlands	373(9.88)	10.6
Lake zone	699(18.52)	19.62
Eastern Zone	541(14.33)	21.32
Zanzibar	311(8.24)	1.3
**Mother’s Education level**		
None	487(12.90)	12.52
Primary education	2,518(66.70)	69.55
Secondary and higher	770 (20.40)	17.93
**Husband’s education level**		
None	225 (7.58)	7.75
Primary	2,084(70.22)	72.19
Secondary and higher	659(22.20)	20.06
**Wealth status**		
Poorest	463(12.26)	12.12
Poorer	587 (15.55)	15.79
Middle	761 (20.16)	19.45
Richer	992 (26.28)	25
Richest	972 (25.75)	27.64
**Occupation**		
Not working	656 (17.38)	18.19
Working	3,119(82.62)	81.81
**Sex of a child**		
Male	1,922(51.56)	51.76
Female	1,806(48.44)	48.24
**Distance to Health facility**		
Not a big problem	2,287(60.58)	58.45
A big problem	1,488(39.42)	41.55

### Reproductive characteristics of the study participants

Reproductive characteristics of the study participants are shown in Table 2. More than half (56%) of the participants attended four ANC visits. Most (92%) of postpartum women had spontaneous vaginal delivery. Majority (88%) reported to have resumed menses. Less than half (44%) of the women reported to have attended postnatal care within two months after birth. About two thirds (62.03%) reported using postpartum contraception for spacing births.

**Table 2:** Reproductive characteristics of the study participants (N = 3775).

Characteristics	n (%)	Weighted %
**Mode of Birth**		
Normal	2,511 (91.98)	91.68
Caesarean	219 (8.02)	8.32
**Place of delivery**		
Public	605 (16.03)	22.45
TBA/Home	1,686 (44.66)	60.97
Private	400 (10.60)	16.58
Missing	1,084(28.72)	
**Number of ANC visits**		
None	30(0.79)	1.34
1–3	1,197 (31.71)	42.30
4+	1,490 (39.47)	56.36
Missing	1,058(28.03)	
**Type of birth**		
Single	3,648 (96.64)	98.11
Multiple	80 (2.12)	1.89
**Postnatal check-up within two months of discharge**		
None	1,172 (31.05)	55.78
yes	900 (23.84)	44.22
Missing	1,703(45.11)	
**Birth order/parity**		
First	733 (19.66)	21.14
2–3	1,357 (36.40)	37.50
4–5	934 (25.05)	23.77
6+	704 (18.88)	17.59
**Exposure to Media**		
No exposure	2,564 (67.92)	67.82
At least one regularly	1,211 (32.08)	32.18
**Empowerment**		
Low	49 (1.30)	3.77
Medium	548 (14.52)	46.81
High	551 (14.60)	49.42
Missing	2,627(69.59)	
**Reasons for FP methods**		
Spacers	2,055 (46.33)	62.03
limiters	1,004 (26.60)	37.97
Missing	1,022(27.07)	
**Type of method opted**		
Short acting methods	1,711 (45.32)	64.65
Long acting Methods	967 (25.62)	35.35
Missing	1,097(29.06)	
**Resumption of menses**		
Not yet resumed	311 (8.24)	11.59
Resumed menses	2,416 (64.00)	88.41
Missing	1,048(27.76)	
**Resumption of sex**		
Resumed sex at 2 months	419 (11.10)	11.05
No resumption at 2 months	3,356 (88.90)	88.95
**Birth delivered by skilled birth attendant**		
No	1373(36.38)	36.6
Yes	2401(63.62)	63.4
**Fertility intention (n = 1,176)**		
Does not intend to use	319(27.13)	27.67
Intends to Use later	857(72.87)	**72.33**
**Breastfeeding**		
Not breastfeeding	2,752 (72.90)	73.60
Current exclusive Breastfeeding	1,023 (27.10)	26.40

### Distribution of contraceptive methods among postpartum women

**Fig 3:**
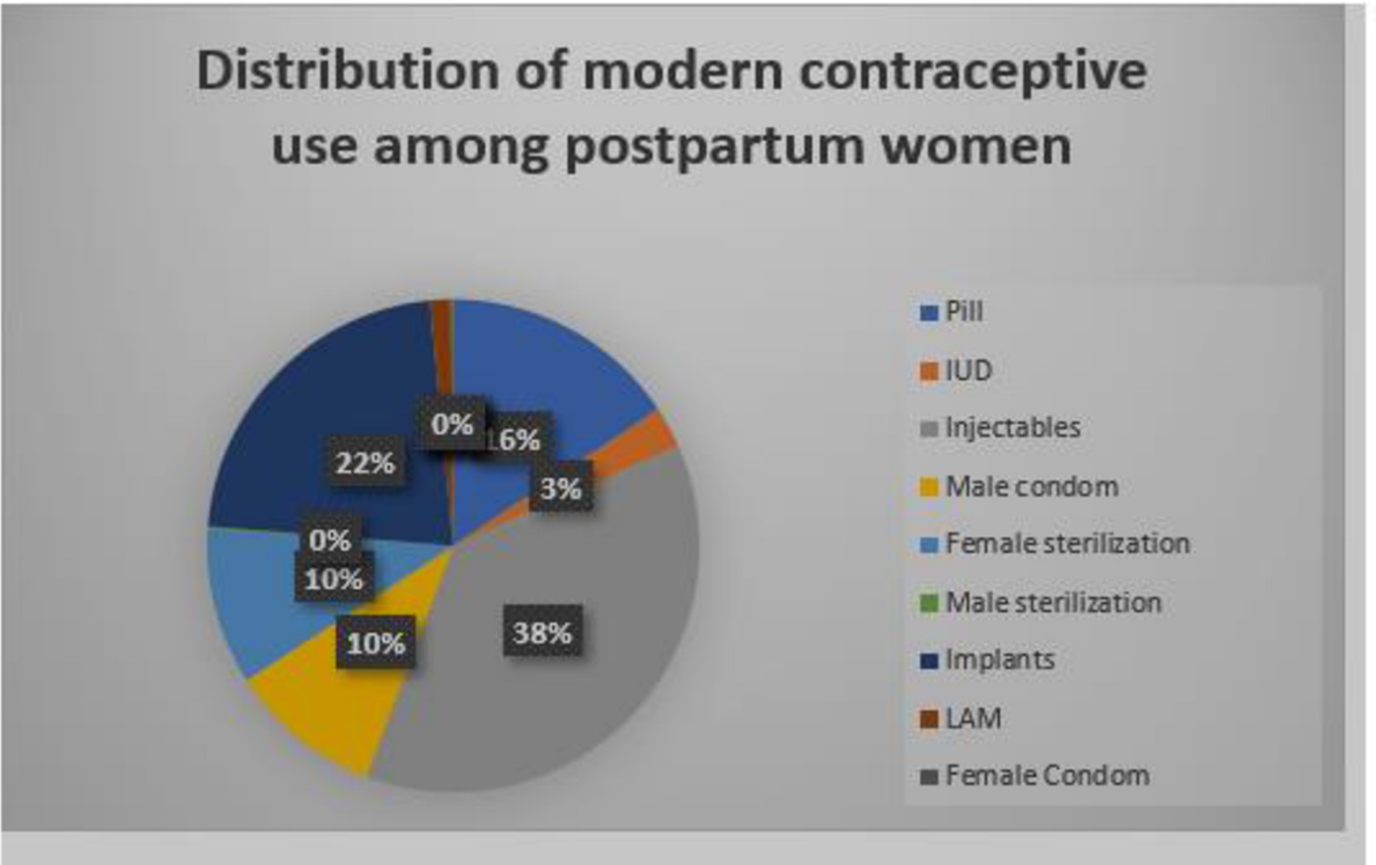
Distribution of modern contraceptive use among postpartum women.

The distribution of contraceptive methods used during postpartum period are showed in Fig 3. The most frequent was injectables (37.39%) followed by implants (22.22%) and pills (15.66%). Other methods were infrequently mentioned.

### Time to resumption of sex after birth

**Fig 4:**
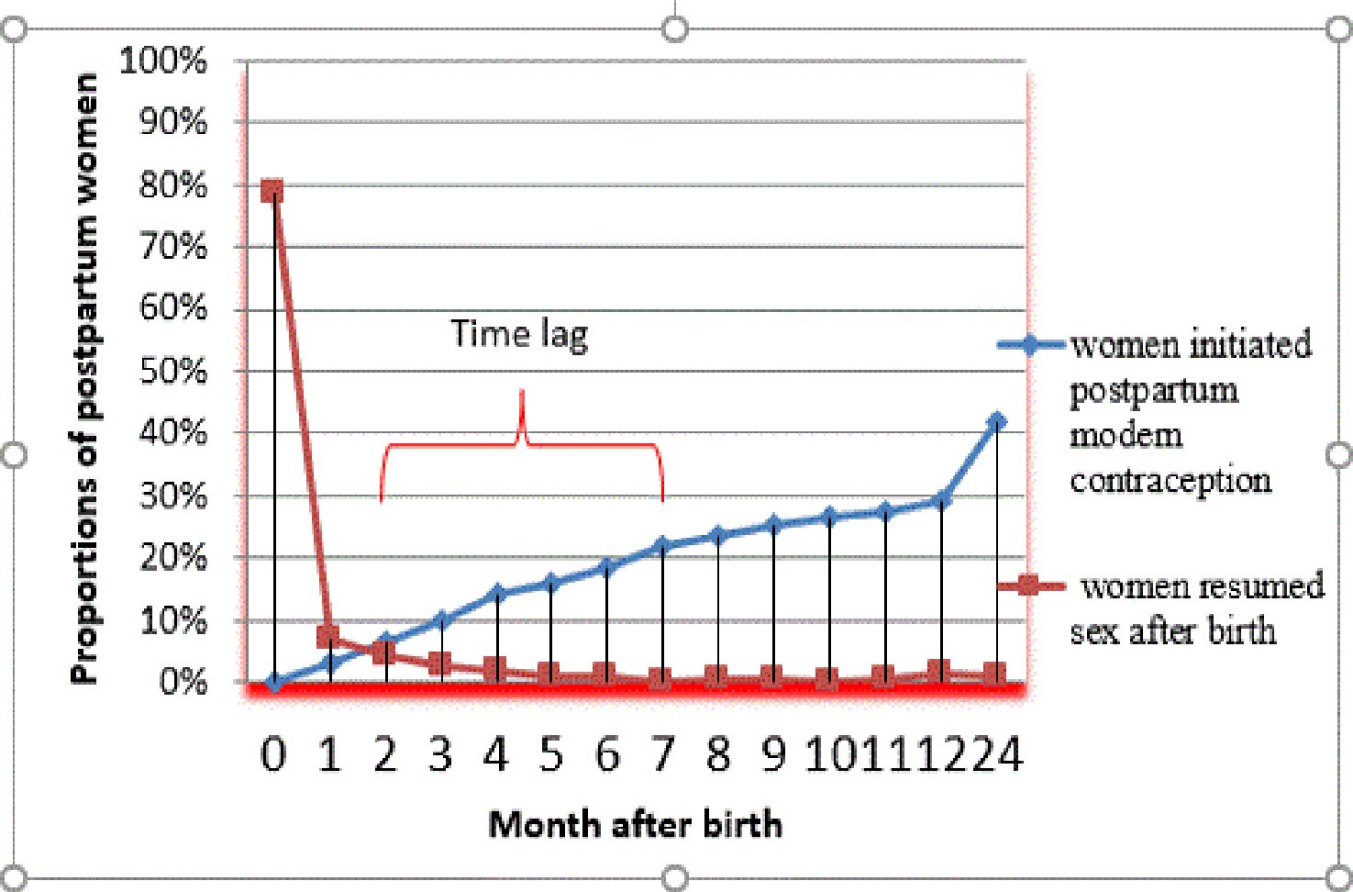
Relationship between resumption to sex and initiation of postpartum contraception.

The relationship between the resumption of sex and initiation of postpartum contraceptive use has been shown in Fig 4. The median time to resumption of sex after birth was 2 (IQR: 1–5) months. Half (50%) of the postpartum women resumed sex earlier as compared to initiation of modern contraception (2 versus 7 months, respectively). This corresponds to 5-month time lag. About 20% of the women initiated modern contraception use at 6 months after birth.

### Time to contraceptive use among postpartum mothers

A total of 1405 (37%) women reported using modern contraception during postpartum period. The median time to contraceptive use after birth was 7(IQR: 4, 13) months. The proportion of women using modern contraceptive use after birth increased over time (Fig 4).

**Fig 5:**
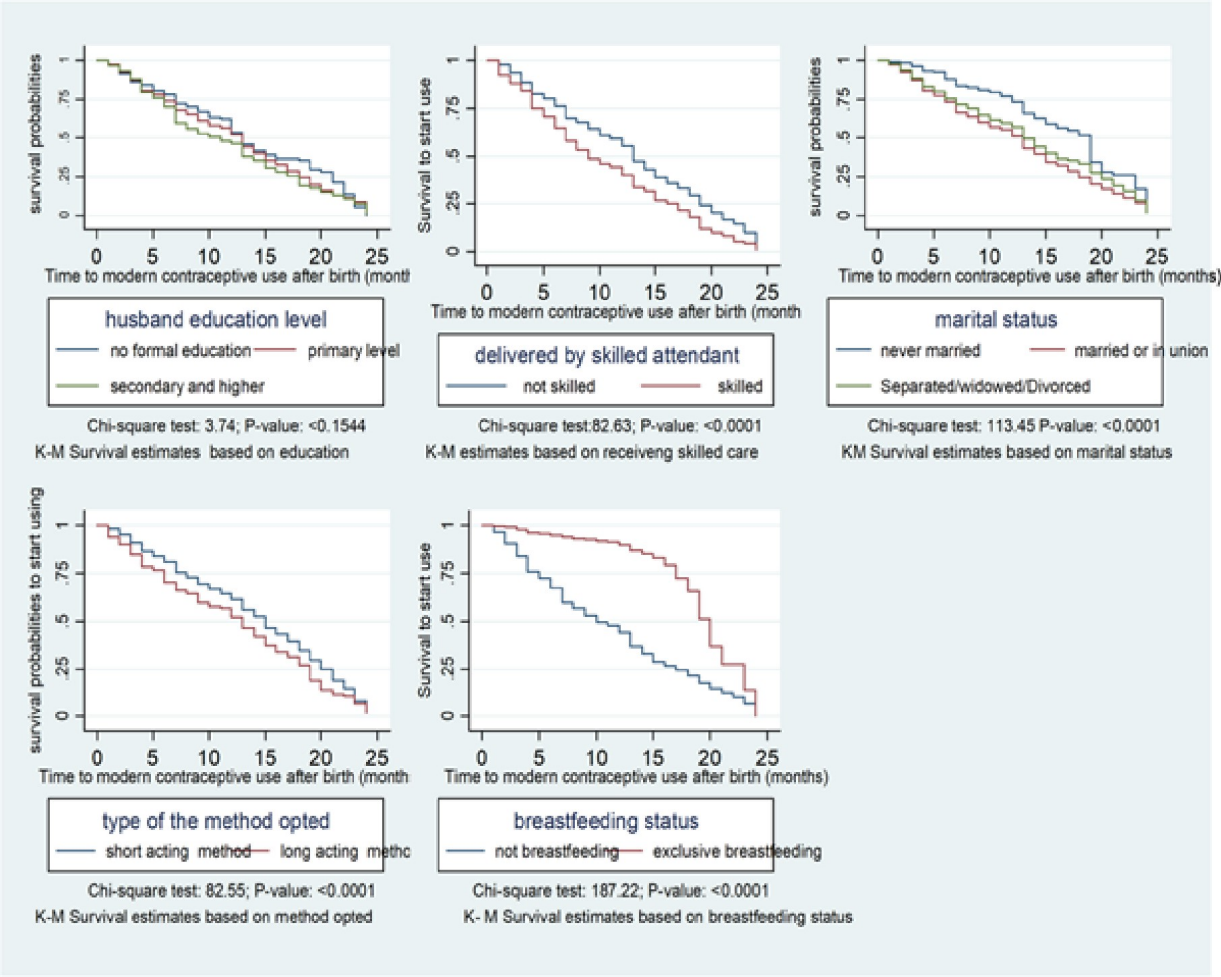
Differentials in time to modern contraceptive use after birth.

The Kaplan Meier (K-M) survival curves demonstrate the relationship between selected participant characteristics and time to postpartum modern contraceptive use (Fig 5). Women who were not married took longer time to initiate modern contraceptive use compared with the ever married counterparts [(12.5 (IQR: 6–19) vs. 7 (IQR: 4, 13) months, respectively] (Table 3). Furthermore, breastfeeding mothers also took longer to initiate postpartum modern contraceptives use compared to their counters. We also noted that women opting for longer acting contraceptives take shorter time to initiate modern contraceptives use compared to those opting the short acting methods. The median time to contraceptive use for women who were never married was found to be12.5 (IQR:6–19) months while married women7(IQR:4,13) and separated women was 9(IQR:4,14.5) months respectively. The differential in time to contraceptive use was assessed using Wilcoxon Log rank test (S1 Table in S1 File).

**Table 3:** Predictors of time to initiate modern contraceptive use after birth Weibull AFT.

Characteristics	Median (Range)	CTR (95%CI)	ATRª(95%CI)
**Age**			
25–29	7(4,13)	Reference	Reference
15–19	7(4,13)	7.88(5.34–11.61)***	1.04(0.72–1.50)
20–24	9(4,15)	1.35(1.12–1.65)**	1.08(0.72–1.63)
30–34	7(4,13)	0.93(0.77–1.13)	0.95(0.59–1.51)
35–39	7(4,13)	1.18(0.96–1.45)	1.00(0.62–1.61)
40–44	6(4,13)	1.54(1.19–1.97)***	0.88(0.51–1.51)
45–49	2(4,9)	3.65(2.28–5.84)***	1.36(0.63–2.90)
**Marital status**			
Married /in union	7(4,13)	Reference	Reference
Never married	13(6,19)	7.62(5.90–9.85)***	1.63(1.26–2.11)**
Separated/widowed/Divorced	9(4,15)	1.45(1.21–1.76)***	1.23(0.99–1.55)
**Residence**			
Urban	6(3,13)	Reference	Reference
Rural	8.5(4,14)	0 .80(0.69–0.93)**	1.02(0.83–1.24)
**Zones**			
Western zone	9(4,13)	Reference	Reference
Northern zone	5.5(3,12)	0.97(0.67–1.38)	0.93(0.67–1.29)
Central zone	7(3,13)	0.99(0 .72–1.37)	0.64(0.45–0.90)
Southern Highlands zone	8(4,13)	0 .92(0 .66–1.28)	0.66(0.51–0.86)
Southern zone	13(7,17)	1.52(1.09–2.11) *	0.70(0.54–0.91)
South West Highlands zone	7(4,14)	0 .99(0.69–1.44)	0.65(0.49–0.85)
Lake zone	8(3,14)	1.02(0.75–1.37)	0.82(0.66–1.02)
Eastern Zone	7(4,13)	1.17(0.85–1.61)	0.78(0.612–0.99)
Zanzibar	4.5(3,10)	0 .69 (0.47–1.01)	0.69(.48-.99)
**Education level**			
None	6(2,12)	Reference	Reference
Primary education	5(1,11)	1.24 (1.03–1.51)**	0.90(0.74–1.09)
Secondary and higher	5(2,10)	2.43(1.89–3.12)**	0.97(0.80–1.17)
**Wealth status**			
Poorest	8(4,14)	Reference	Reference
Poorer	10(5,15)	1.02(0.80–1.28)	1.02(0.80–1.29)
Middle	8(4,14)	1.11(0.88–1.41)	1.06(0.83–1.35)
Richer	7(3,13)	1.12(0.88–1.42)	0.84(0.65–1.09)
Richest	6(3,13)	1.44(1.12–1.84)**	0.73(0.54–0.99)*
**Working status**			
Not working	7(4,13)	Reference	Reference
Working	7(4,14)	0.62(0.50–0.76)**	0.98(0.81–1.19)
**Place of delivery**			
Institutions	6(3,12)	Reference	Reference
Non institution	7(4,13)	1.10(0.98–1.24)	0.93(0.77–1.12)
**Number of ANC visits**			
Less than 4 visits	7(3,13)	Reference	Reference
Recommended	6(3,12)	0.95(0.86–1.04)	0.93(0.81–1.08)
**Breastfeeding**			
No breastfeeding	7(4,13)	Reference	Reference
Breastfeeding	8(4,16)	3.75(2.79–5.04)***	5.50(4.12–7.40) **
**Postnatal check up within two months of discharge**			
None	6(3,12)	Reference	Reference
Yes	6(3,2)	0.94(0.83–1.07)	0.98(0.84–1.17)
**Birth delivered by skilled birth attendant**			
No	7(1,14)	Reference	Reference
Yes	6(3,12)	0.38(0.32–0.46)***	0.92(0.75–1.12)
**Birth order/parity**			
First	8(5,14)	Reference	Reference
2–3	7(4,13)	0.99(0.85–1.17)	1.01(0.78–1.29)
4–5	7(4,14)	1.01(0.86–1.19)	0.91(0.73–1.15)
6+	7(3,13)	1.27(1.05–1.52)*	1.06(0.77–1.44)
**Exposure to Media**			
No exposure	7(4,13)	Reference	Reference
At least one regularly	7(4,14)	0.81(0.69–0.93)**	1.02(0.87–1.19)
**Purpose of use**			
Spacers	8(4,15)	Reference	Reference
Limiters	6(3,13)	0.82(0.67–0.98)*	1.02(0.85–1.21)
**Type of method opted**			
Short acting methods	8(4,15)	Reference	Reference
Long acting Methods	6(3,13)	0.45(0.37–0.53)***	0.70(0.60–0.82)***
**Resumption of menses**			
Not yet resumed	7(4,13)	Reference	Reference
Resumed menses	7(3,13)	0.66(0.51–0.87) **	0.82(0.56–1.24)
**Resumption of sex**			
Did not resume sex by 2^nd^ month	7(4,15)	Reference	Reference
Had resumed sex by 2^nd^ month	7(4,13)	0.49(0.36–0.66)**	1.11(0.82–1.51)

Adjusted for partner age difference, husband’s highest education level, mode of birth, sex of a child, type of birth.

cTR: crude time ratios.

aTR: adjusted time ratios.

*Effect was statistically significant at 0.05,

**Effect was statistically significant at 0.001,

***Effect was statistically significant at 0.001.

### Predictors of time to initiate postpartum modern contraceptive use

The crude and adjusted estimates for predictors for time to modern postpartum contraceptive use are showed in Table 3. After adjusting for other factors, we found that at any point in time never married women took 63% expected longer time to initiate postpartum modern contraception compared with married women (TR: 1.63; 95%CI: 1.26–2.11). Women from the richest quintile had 27% expected shorter time to initiate postpartum modern contraception compared to women in the poorest wealth quintile (TR: 0.73; 95%CI: 0.54–0 .99). Women who practiced exclusive breastfeeding had 6-fold longer time to initiate postpartum modern contraceptives compared to no-breastfed counterparts (TR: 5.50; 95% CI: 4.12–7.35) (Table 3) Similarly, women who opted for longer method of contraception had 30% lesser time to adopt postpartum modern contraception compared to their counterparts who opted to use shorter methods (TR:0.70;95%CI: 0.60–0.82).

## Discussion

The median time to postpartum modern contraceptive use was 7 months while the median time to resume sex after birth was 2 months. Time to contraceptive use was longer among unmarried compared to married and ever married women (13 versus 7 versus 9) respectively. It was observed to be longer for women who were spacing compared to women limiting births (8 versus 6) months respectively. This is consistent with previous studies [27–30] which reported median time to contraceptive use ranging between 6–8 months. The similarity in finding between our study and other studies could be explained by the fact that both were observational studies using a calendar as a tool for collection.

The finding poses five months lag period for postpartum women to be exposed to the risk of unintended pregnancy. The evidence demonstrates that proportionately large number of these women would be at risk of unplanned pregnancy and sub optimal IPI due to delays in adoption of methods after birth considering the high levels of unmet need in this group of women in Tanzania and suboptimal practices of breastfeeding [9, 31]. Hence forth it suggests that contraception counseling initiated prior to postpartum discharge and reinforced at any time within 6 weeks as recommendation is critical to avert the risk of unintended pregnancy and associated outcomes.

Regarding the household wealth, the study identified that women from richest wealth quintile took shorter median time to postpartum modern contraceptives use compared to the poorest. This finding is consistent with previous studies [15, 32, 33] suggesting that economically empowered women have ability to do household purchases and even seek health care with reduced barriers as decision will not depend on finances from her partner [34]. Despite contraceptives being universal indirect costs such as transport to the facility and actual money to buy for dual methods have to be catered by the user. The study highlights the need to empower postpartum women through economic opportunities to be able to make informed choices early.

In this study, being married/cohabiting was associated with expected short time to contraceptive use compared to unmarried counterparts. This was consistent with previous studies [32, 34] whereby could be explained by the difference in preference, needs and possible awareness about the implication of unprotected coitus among women across their marital statuses. It might be that sexual intercourse among never married women is sporadic as compared to married ones and hence ignoring use of modern methods [35]. Following this delays to use among separated/unmarried women, it is not surprising that the unmet need has remained to be high to this group [35]. Previous studies have paid much focus on married women their sexual activity and contraceptive use, leaving out this unmarried group may back-track the significant progress on reproductive health due to risks associated with unintended pregnancy. The potential social, economic and health-related consequences of unintended pregnancy for never married women make it essential to address the identified delay for postpartum modern contraception.

Women who opted for longer method of contraception took 30% times lesser time to adopt postpartum modern contraception compared to ones opted for shorter methods. This finding is consistent with previous study which was conducted elsewhere [36] who revealed that long acting methods were two times likely to be initiated in early postpartum compared to others. The findings imply method effectiveness had strong attribute for early initiation of postpartum contraception among women in Tanzania. Also would be on account that elective caessarian poses opportunity for a health care provider to counsel the client and ensure its availability at the time of delivery for adoption. Our finding suggests a need for early contraceptive counseling to be tailored to the client preferences to enable early adoption of the opted method.

Furthermore in the current study, women residing in southern west highland zones had 30% shorter times to initiate postpartum modern contraceptive use. This is consistent with other studies [15, 33] who found that a place of residence was a significant predictor for early timing of postpartum modern contraception. It emphasizes the need to close disparities in access between the zones by scaling up long acting methods and sustaining efforts to universal accessibility of methods of modern contraception especially in rural areas.

### Strengths and weakness

The large sample size of this study imply having high power of the study to increase ability to assess the true effect between predictors and time to modern contraceptive use among postpartum women. DHS use of the contraceptive calendar data, this added considerable value to the analysis in terms of verifying the consistency in sequencing of reproductive events (i.e pregnancy, births and subsequent contraceptive use), thereby minimizing the effect of recall bias. A weibull AFT model added a value in this study as it estimated the expected survival times between group categories in time ratios. An AFT model does not require PH assumption (i.e strong assumption) as the Cox regression does and it is more appealing than the PH models because of its quite direct physical interpretation. Further the estimated regression parameters in AFT models are robust against the misspecification of the model assumption [26, 37].

Caution should be taken while interpreting these results. Using secondary data source is subject to missing of important information eg birth related morbidity and method availability which could lead to residual confounding (unmeasured confounding) which might result to over or under estimate the effect measure in the study. The possibility of reporting bias during data collection might be a problem as some participants might have not remembered accurately the solicited information especially on short acting methods like condom and richest wealth quintile might have been overrepresented. Also since DHS data are collected on monthly basis on a calendar, this makes difficult to delineate the post placental and immediate postpartum use as all were represented by month one.

## Conclusions

In this study, the median time to contraceptive use was 7 months. Numerous factors were associated with time to modern contraceptive use. This include being resident to southern west zone, having never been married, belonging to richest wealth quintile, practicing recommended breastfeeding for six months as well as adopting long acting method. Policy makers and programmers need to strengthen efforts promoting early initiation of postpartum contraception as an endeavor to adhere to appropriate 6 week recommendation of postpartum family planning in trying to curb down the 5month lag period identified. They should be informed of short time and longtime predictors for interceptive measures for timely availing of the methods and for investments to increase modern Contraceptive Prevalence Rate (mCPR) in postpartum period. We recommend interventions specific targeting unmarried women to promote early adoption of postpartum contraception. Furthermore, future qualitative study to be conducted to understand the group of never married women and their perceptions, knowledge and practices towards timely adoption of modern contraception.

## Supporting information

S1 File (ZIP)

## Abbreviations

ANC: antenatal care
HSSP: Health Sector Strategic Plan
KCMUCo: Kilimanjaro Christian Medical University college
MoHCDGEC: Ministry of Health Community Development, Gender, Elderly and Children
PMTCT: Prevention of Mother to Child Transmission of HIV
PPFP: postpartum Family Planning
TDHS-MIS: Tanzania Demographic and health survey and Malaria Impact Survey
TR: Time ratio

## References

[1] Gaffield ME, Egan S, Temmerman M. It’s about time: WHO and partners release programming strategies for postpartum family planning. Glob Heal Sci Pract. 2014;2(1):4–9. 10.9745/GHSP-D-13-00156 25276558 PMC4168603

[2] Ahmed S, Li Q, Liu L, Tsui AO. Maternal deaths averted by contraceptive use: An analysis of 172 countries. Lancet [Internet]. 2012;380(9837):111–25. Available from: 10.1016/S0140-6736(12)60478-4 22784531

[3] Stover J, Ross ÆJ. How Increased Contraceptive Use has Reduced Maternal Mortality. 2010;687–95. 10.1007/s10995-009-0505-y 19644742

[4] Izugbara C, Population A, Wekesah F, Population A, Tilahun T, Amo-adjei J. Family Planning in East Africa: Trends and Dynamics Family Planning in East Africa: Trends and Dynamics. 2018;(January).

[5] United Nations. Trends in Contraceptive Use Worldwide [Internet]. New York; 2015. Available from: www.unpopulation.org

[6] Track20 project. Trends in the Uptake of Postpartum Family Planning [Internet]. PPFP. 2017. Available from: http://www.track20.org/pages/data_analysis/in_depth/PPFP/trends.php

[7] MOHCDGEC. United republic of tanzania the national road map strategic plan to improve health in Tanzania (2016–2020). 2016;ONEPLAN II(June 2016):12–25.

[8] MOHCDGEC. Health Sector Strategic Plan. 2015;HSSP IV(August,2015):43.

[9] Mcsp. Family Planning Needs during the First Two Years Postpartum in Tanzania. 2015;13 (September 2008):3–7.

[10] WHO. Programming strategies for Postpartum Family Planning [Internet]. 1st Editio. World Health Organization, editor. Vol. 22, Programming Strategies for Postpartum Family Planning. WHO Press; 2013. 294–307 p. Available from: www.who.int

[11] MoHCDGEC. National Family Planning Guidelines and Standards. 2013. 15–64 p.

[12] WHO. Contraceptive prevalence [Internet]. Sexual and reproductive health. 2018 [cited 2018 Sep 9]. Available from: http://www.who.int/reproductivehealth/topics/family_planning/contraceptive_prevalence/en/

[13] Owonikoko K, Adeoye A, Tijani A, Adeniji A. Determinants of resumption of vaginal intercourse in puerperium period in Ogbomoso: consideration for early use of contraceptives. Int J Reprod Contraception, Obstet Gynecol [Internet]. 2014;3(4):1061. Available from: http://www.ijrcog.org/index.php/ijrcog/article/view/2230

[14] Bello FA, Olayemi O, Aimakhu CO, Adekunle AO. Effect of Pregnancy and Childbirth on Sexuality of Women in Ibadan, Nigeria. ISRN Obstet Gynecol [Internet]. 2011;2011:1–6. Available from: http://www.hindawi.com/journals/isrn/2011/856586/ 10.5402/2011/856586 21647230 PMC3101881

[15] Padmadas SS, Lyons-amos M, Thapa S. Contraceptive behavior among women after abortion in Nepal ☆. Int J Gynecol Obstet [Internet]. 2014;127(2):132–7. Available from: 10.1016/j.ijgo.2014.05.012 25047427

[16] Winfrey W, Rakesh K. Use of Family Planning in the Postpartum Period: Dhs Comparative Report. ICF international. Rockville, Maryland, USA:; 2014.

[17] Hounton S, Winfrey W, Barros AJD, Askew I, Hounton S, Winfrey W, et al. Patterns and trends of postpartum family planning in Ethiopia, Malawi, and Nigeria: evidence of missed opportunities for integration. 2015;9716. 10.3402/gha.v8.29738 PMC4642460 26562144

[18] Wamala R, Kabagenyi A, Kasasa S. Predictors of Time-to-Contraceptive Use from Resumption of Sexual Intercourse after Birth among Women in Uganda.

[19] Mushy SE, Tarimo EAM, Fredrick Massae A, Horiuchi S. Barriers to the uptake of modern family planning methods among female youth of Temeke District in Dar es Salaam, Tanzania: A qualitative study. Sex Reprod Healthc [Internet]. 2020;24(June 2019):100499. Available from: 10.1016/j.srhc.2020.100499 32050123

[20] Yazdkhasti M, Pourreza A, Pirak A. Unintended Pregnancy and Its Adverse Social and Economic Consequences on Health System: A Narrative Review Article. 2015;44(1):12–21. PMC4449999 26060771

[21] Zhang Q, Dang S, Bai R, Mi B, Wang L, Yan H. Association between maternal interpregnancy interval after live birth or pregnancy termination and birth weight: A quantile regression analysis. Sci Rep [Internet]. 2018;8(1):1–8. Available from: 10.1038/s41598-018-22498-0 29515137 PMC5841397

[22] Mahande MJ, Obure J. Effect of interpregnancy interval on adverse pregnancy outcomes in northern Tanzania: A registry-based retrospective cohort study. BMC Pregnancy Childbirth [Internet]. 2016;16(1):1–9. Available from: 10.1186/s12884-016-0929-5 27268015 PMC4897820

[23] MoHCDGEC, MoH, NBS, OCGS and I. Tanzania. Tanzania Demogr Heal Surv Malar Indic syrvey 2015–16. 2016;5–39.

[24] DHS. DHS Contraceptive Calendar Tutorial [Internet]. 2nd ed. ICF Internationa, editor. 530 Gaither Road, Suite 500, Rockville MD, 20850, USA: USAID); 2018. 4–48 p. Available from: www.DHSprogram.com

[25] Orbe J, Ferreira E, Nã V. Comparing proportional hazards and accelerated failure time models for survival analysis. 2002;3510(January 2001):3493–510. 10.1002/sim.1251 12407686

[26] Faruk A. The comparison of proportional hazards and accelerated failure time models in analyzing the first birth interval survival data. J Phys Conf Ser. 2018;974(1).

[27] Ndugwa RP, Cleland J, Madise NJ, Fotso JC, Zulu EM. Menstrual pattern, sexual behaviors, and contraceptive use among postpartum women in Nairobi Urban Slums. J Urban Heal. 2011;88(SUPPL. 2). 10.1007/s11524-010-9452-6 PMC3132236 20449772

[28] Huang Y, Merkatz R, Kang J, Roberts K, Hu X, Di F, et al. Postpartum unintended pregnancy and contraception practice among rural-to-urban migrant women in Shanghai. Contraception [Internet]. 2012;86(6):731–8. Available from: 10.1016/j.contraception.2012.05.007 22703950

[29] Do M, Hotchkiss D. Relationships between antenatal and postnatal care and post-partum modern contraceptive use: evidence from population surveys in Kenya and Zambia. 2013; 10.1186/1472-6963-13-6 PMC3545900 23289547

[30] Keogh SC, Urassa M, Kumogola Y, Kalongoji S, Kimaro D, Zaba B. Postpartum Contraception in Northern Tanzania: Patterns of Use, Relationship to Antenatal Intentions, and Impact of Antenatal Counseling. 2015;405–22. 10.1111/j.1728-4465.2015.00040.x 26643490

[31] Kazaura M. Exclusive breastfeeding practices in the Coast region, Tanzania. Exclus Breastfeed Pract Coast Reg Tanzania Afri Heal Sci 2016;16(1) 44–50 http//dx.doi org/104314/ahs.v16i16 Introd. 2016;16(1):44–50. 27358612 10.4314/ahs.v16i1.6 PMC4915437

[32] Fagbamigbe AF, Adebowale AS, Morhason-Bello I. Survival analysis of time to uptake of modern contraceptives among sexually active women of reproductive age in Nigeria. BMJ Open. 2015;5(12):1–11. 10.1136/bmjopen-2015-008371 26671948 PMC4679946

[33] Wamala R, Kabagenyi A, Kasasa S. Predictors of Time-to-Contraceptive Use from Resumption of Sexual Intercourse after Birth among Women in Uganda. 2017;2017:7–10.

[34] Jalang’O R, Thuita F, Barasa SO, Njoroge P. Determinants of contraceptive use among postpartum women in a county hospital in rural Kenya. BMC Public Health. 2017;17(1):1–8. 10.1186/s12889-016-3954-4 28662695 PMC5492366

[35] Sedgh G, Ashford LS, Hussain R. Unmet Need for Contraception in Developing Countries: Examining Women’s Reasons for Not Using a Method. Guttmacher Inst. 2016;(June):2–48.

[36] van Schalkwyk C. Postpartum Insusceptibility to Pregnancy and Modern Contraceptive Use in Nepal and Tanzania Erin Pearson, Postdoctoral Research Fellow, Harvard T.H. Chan School of Public Health. 2015;1–16.

[37] Alfensi F. The comparison of proportional hazards and accelerated failure time models in analyzing the first birth interval survival data The comparison of proportional hazards and accelerated failure time models in analyzing the first birth interval survival data. 2018.

