# Supplementary material for: Initiation of postpartum modern contraceptive methods: Evidence from Tanzania demographic and health survey
Source: PLoS One. 2021 Mar 25;16(3):e0249017. doi: 10.1371/journal.pone.0249017 (PMC7993875; doi:10.1371/journal.pone.0249017)
Supplement: S1 File — (ZIP) [file pone.0249017.s001.zip › New folder/Appendix.docx]

**Appendix**

**Table4.Differentials in time-to-contraceptive use based on Wilcoxon Log rank Chi-square test**

| **Characteristic** | **χ²** | **P value** |
| --- | --- | --- |
| Age | 100.81 | <0.001 |
| Male-female age difference | 3.26 | 0.3528 |
| Marital status | 113.45 | <0.0001 |
| Residence | 4.95 | 0.0261 |
| Zones | 59.31 | <0.001 |
| Education level | 9.13 | 0.0104 |
| Husband’s highest education level | 3.74 | 0.1544 |
| Wealth status | 13.87 | 0.0077 |
| Working status | 1.62 | 02.008 |
| Mode of Birth | 10.12 | 0.0015 |
| Place of delivery | 8.93 | 0.0115 |
| Number of ANC visits | 9.38 | 0.0092 |
| Type of birth | 1.45 | 0.2290 |
| Sex of a child | 0.95 | 0.3370 |
| Breastfeeding | 187.22 | <0.0001 |
| Postnatal checkup within two months of discharge | 4.10 | 0.0428 |
| Birth order/parity | 5.99 | 0.1120 |
| Exposure to Media | 1.75 | 0.1854 |
| Empowerment | 0.98 | 0.6126 |
| Purpose of use | 2.60 | 0.1068 |
| Type of method opted | 69.32 | <0.001 |
| Specific methods used | 176.28 | <0.001 |
| Resumption of menses | 25.19 | <0.001 |
| Resumption of sex | 11.07 | 0.0009 |
| Distance | 1.71 | 0.1909 |

**χ²: Wilcoxon Log rank test Chi square**
