# Supplementary material for: Initiation of postpartum modern contraceptive methods: Evidence from Tanzania demographic and health survey
Source: PLoS One. 2021 Mar 25;16(3):e0249017. doi: 10.1371/journal.pone.0249017 (PMC7993875; doi:10.1371/journal.pone.0249017)
Supplement: S1 File — (ZIP) [file pone.0249017.s001.zip › New folder/Fig 1 Conceptual framework.docx]

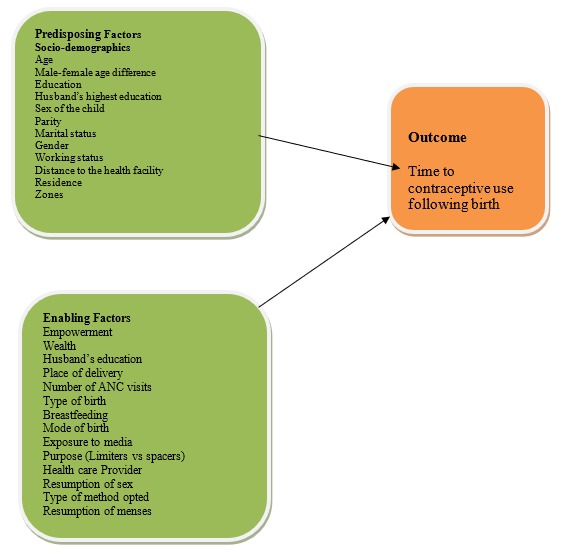


Figure 1 Conceptual framework on predictors of time to initiate postpartum contraceptive. On the left-hand side of the framework lists socio-demographic and enabling factors predisposing to prediction of time to initiate postpartum contraceptive use.
