# Supplementary figures and images for: Initiation of postpartum modern contraceptive methods: Evidence from Tanzania demographic and health survey
Source: PLoS One. 2021 Mar 25;16(3):e0249017. doi: 10.1371/journal.pone.0249017 (PMC7993875; doi:10.1371/journal.pone.0249017)

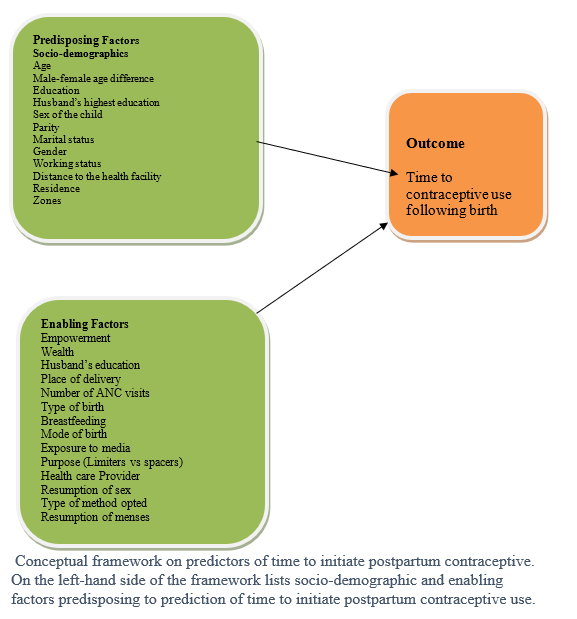

Supplement: S1 File — (ZIP) [file pone.0249017.s001.zip › New folder/conceptual frame.tif]

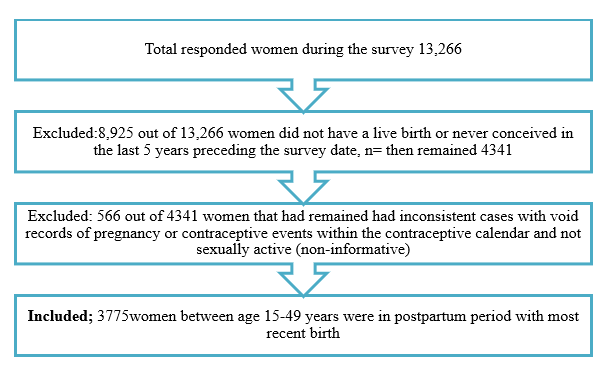


Figure 2. Schematic diagram for the sample size estimation (n=3775)

Supplement: S1 File — (ZIP) [file pone.0249017.s001.zip › New folder/Schematic diagram.docx]

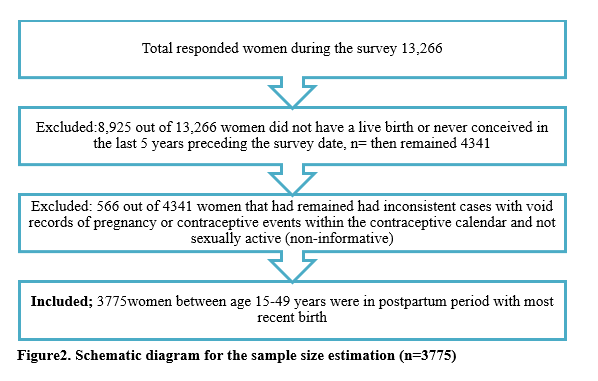

Supplement: S1 File — (ZIP) [file pone.0249017.s001.zip › New folder/schematic_diagram.tif]
